# Broadly protective immunity against divergent influenza viruses by oral co-administration of *Lactococcus lactis* expressing nucleoprotein adjuvanted with cholera toxin B subunit in mice

**DOI:** 10.1186/s12934-015-0287-4

**Published:** 2015-08-05

**Authors:** Han Lei, Xiaojue Peng, Huifeng Jiao, Daxian Zhao, Jiexiu Ouyang

**Affiliations:** College of Medicine, Southwest Jiaotong University, Chengdu, 610031 Sichuan China; Department of Biotechnology, College of Life Science, Nanchang University, Nanchang, 330031 Jiangxi China

**Keywords:** *L. lactis*/pNZ8008-NP adjuvanted with CTB, Cross-protective immunity, Influenza A viruses

## Abstract

**Background:**

Current influenza vaccines need to be annually reformulated to well match the predicated circulating strains. Thus, it is critical for developing a novel universal influenza vaccine that would be able to confer cross-protection against constantly emerging divergent influenza virus strains. Influenza virus A is a genus of the Orthomyxoviridae family of viruses. Influenza virus nucleoprotein (NP) is a structural protein which encapsidates the negative strand viral RNA, and anti-NP antibodies play role in cross-protective immunity. *Lactococcus lactis* (*L. lactis*) is an ideal vaccine delivery vehicle via oral administration route. However, *L. lactis* vectored vaccine exhibits poor immunogenicity without the use of mucosal adjuvant. To enhance the immunogenicity of *L. lactis* vectored vaccine, cholera toxin B (CTB) subunit, one of mucosal adjuvants, is a safe adjuvant for oral route, when combined with *L. lactis* vectored vaccine. In this study, we hypothesized that pNZ8008, a *L. lactis* expression plasmid, encoding NP antigen, would be able to elicit cross-protection with the use of CTB via oral administration route.

**Results:**

To construct *L. lactis* vectored vaccine, nucleoprotein (NP) gene of A/California*/*04*/*2009*(*H1N1) was sub-cloned into a *L. lactis* expression plasmid, pNZ8008. The expression of recombinant *L. lactis*/pNZ8008-NP was confirmed by Western blot, immunofluorescence assay and flow cytometric analysis. Further, immunogenicity of *L. lactis*/pNZ8008-NP alone or adjuvanted with cholera toxin B (CTB) subunit was evaluated in a mouse model via oral administration route. Antibodies responses were detected by ELISA. The result indicated that oral administration of *L. lactis*/pNZ8008-NP adjuvanted with CTB could elicit significant humoral and mucosal immune responses, as well as cellular immune response, compared with *L. lactis*/pNZ8008-NP alone. To further assess the cross-protective immunity of *L. lactis*/pNZ8008-NP adjuvanted with CTB, we used *L. lactis*/pNZ8110-pgsA-HA1 alone or adjuvanted with CTB as controls. Mice that received *L. lactis*/pNZ8008-NP adjuvanted with CTB were completely protected from homologous H1N1 virus and showed 80% protection against heterologous H3N2 or H5N1 virus, respectively. By contrast, *L. lactis*/pNZ8110-pgsA-HA1 adjuvanted with CTB also conferred 100% protection against H5N1 virus infection, but indicated no cross-protection against H1N1 or H5N1 virus challenge. As controls, mice vaccinated orally with *L. lactis*/pNZ8008-NP alone or *L. lactis*/pNZ8110-pgsA-HA1 alone could not survive.

**Conclusion:**

This study is the first to report the construction of recombinant *L. lactis*/pNZ8008-NP and investigate its immunogenicity with the use of CTB. Compared with *L. lactis*/pNZ8110-pgsA-HA1 adjuvanted with CTB, our data support 5 × 10^11^ CFU of *L. lactis*/pNZ8008-NP adjuvanted with 1 µg of CTB is a better combination for universal influenza vaccines development that would provide cross-protective immunity against divergent influenza A viruses.

## Background

Influenza A viruses have a great threat to public health concern and cause significant morbidity and mortality worldwide [1]. Seasonal influenza H1N1 and H3N2 viruses cause an average of 3–5 million cases of severe illness and up to 250,000–500,000 deaths annually [2]. Additionally, World Health Organization (WHO) has reported that total of 666 cases infected with H5N1 causing 393 death in humans, since 2003 [3].

Vaccination is the most effective way to prevent and control influenza A viruses [1, 4]. Most of currently available influenza vaccines focus on the induction of neutralizing antibodies that target the surface viral protein hemagglutinin (HA), which is subjected to a high degree of antigenic variation and new divergent strains continuously arise in nature [5]. In this case, influenza vaccines need to be updated annually to well match the predicted virus strains [1]. Therefore, this brings out an urgent need for the development of an influenza vaccine that would be able to protect against the multiple antigenic variants.

It is well known that the development of a universal influenza vaccine relies on highly conserved regions of antigens [6, 7], such as the stalk domain of the HA [8, 9], the amino terminus of the M2 proteins (M2e or M2 ectodomain) [10] and the nucleoprotein (NP) [11]. Of note, phylogenetic analysis of virus strains isolated from different hosts indicates that the NP is relatively well conserved [12]. In addition, Influenza virus nucleoprotein (NP) is a structural protein which encapsidates the negative strand viral RNA [12]. Therefore, a highly conserved NP protein is an attractive candidate for a broad-spectrum influenza vaccine [7, 12–14]. Furthermore, the NP protein is the major target antigen for cytotoxic T lymphocyte (CTL) responses, and elicits cross-protective immunity to speed up viral clearance [15–18]. Usually, mucosal immunization (oral or intranasal) is not sufficient for eliciting strong antibodies responses without the use of mucosal adjuvant. cholera toxin B (CTB) has been used successfully as a safe mucosal adjuvant with oral administration [19]. Importantly, CTB can stimulate upregulation on antigen-presenting cells (APCs) and promote Th2 immune responses [20]. To investigate the cross-protection of NP, Tamura et al., developed recombinant NP protein based on baculovirus expression system, and then combined with the adjuvant cholera toxin B subunit (CTB) via intranasal immunization in the mouse model, which indicated that 70–80% of mice were protected from homologous challenge with A/Puerto Rico/8/1934 (H1N1) and 40–70% of mice were protected from heterologous challenge [21]. However, most of effective approaches based on viral vectors, such as adenovirus [22] and baculovirus [21] expressing NP protein, are associated with potential safety issues [23].

Mucosal vaccination can provide the first line of defense against the entry of influenza virus. Thus, important features of a novel influenza vaccine would be its capacity to induce strong mucosal immune response, as well as humoral and cellular immune responses [24–26]. *Lactococcus lactis* (*L. lactis*) is a Gram-positive bacterium and therefore does not possess endotoxic lipopolysaccharides (LPS) [27]. Importantly, it is generally regarded as safe (GRAS) that is used for centuries in the food industry [27]. These features lead to *L. lactis* is suitable for mucosal immunization as a vaccine delivery platform. Our previous studies have reported that the HA1 protein of avian influenza H5N1 virus was displayed on *L. lactis* surface (*L. lactis*/pNZ8110-pgsA-HA1), mice co-vaccinated orally with recombinant *L. lactis*/pNZ8110-pgsA-HA1 adjuvanted with CTB were completely protected from homologous H5N1 virus infection [19]. Further, we extended our work that intranasal vaccination of *L. lactis*-HA adjuvanted with labile enterotoxin B (LTB) subunit could also confer 100% protection against homologous H5N1 virus infection in the chicken model [28]. However, influenza vaccines that provide heterotypic protection are the increasing concerns [1]. Due to NP protein has cross-reactivity and shows poor immunogenicity [12], we hypothesized that recombinant *L. lactis* expressing NP protein with the use of a mucosal adjuvant would be able to provide cross-protective immunity against divergent influenza A viruses.

To address this hypothesis, we investigated the immunogenicity of *L. lactis* expressing NP protein adjuvanted with a mucosal adjuvant, CTB, in a mouse model. Mice co-administered orally with recombinant *L. lactis/*pNZ8008-NP adjuvanted with CTB elicited significant humoral and cellular immune responses, as well as mucosal immune response, compared with oral administration of *L. lactis/*pNZ8008-NP alone. In this study, H1N1, H3N2 and H5N1 are chosen for virus challenge, since they are generally used in developing influenza vaccine that provides cross-protection. Expectedly, *L. lactis/*pNZ8008-NP adjuvanted with CTB provided cross-protective immunity against homologous H1N1 virus and heterologous H5N1 or H3N2 virus, compared with *L. lactis*/pNZ8110-pgsA-HA1 adjuvanted with CTB that showed complete protection against homologous H5N1 virus and no cross protection against heterologous H1N1 or H3N2 virus. Therefore, *L. lactis/*pNZ8008-NP adjuvanted with CTB is an ideal combination for developing a novel influenza vaccine that confers cross-protective immunity against divergent influenza A viruses.

## Results

### Construction of *L. lactis* vectored vaccine and expression of NP protein

**Fig. 1:**
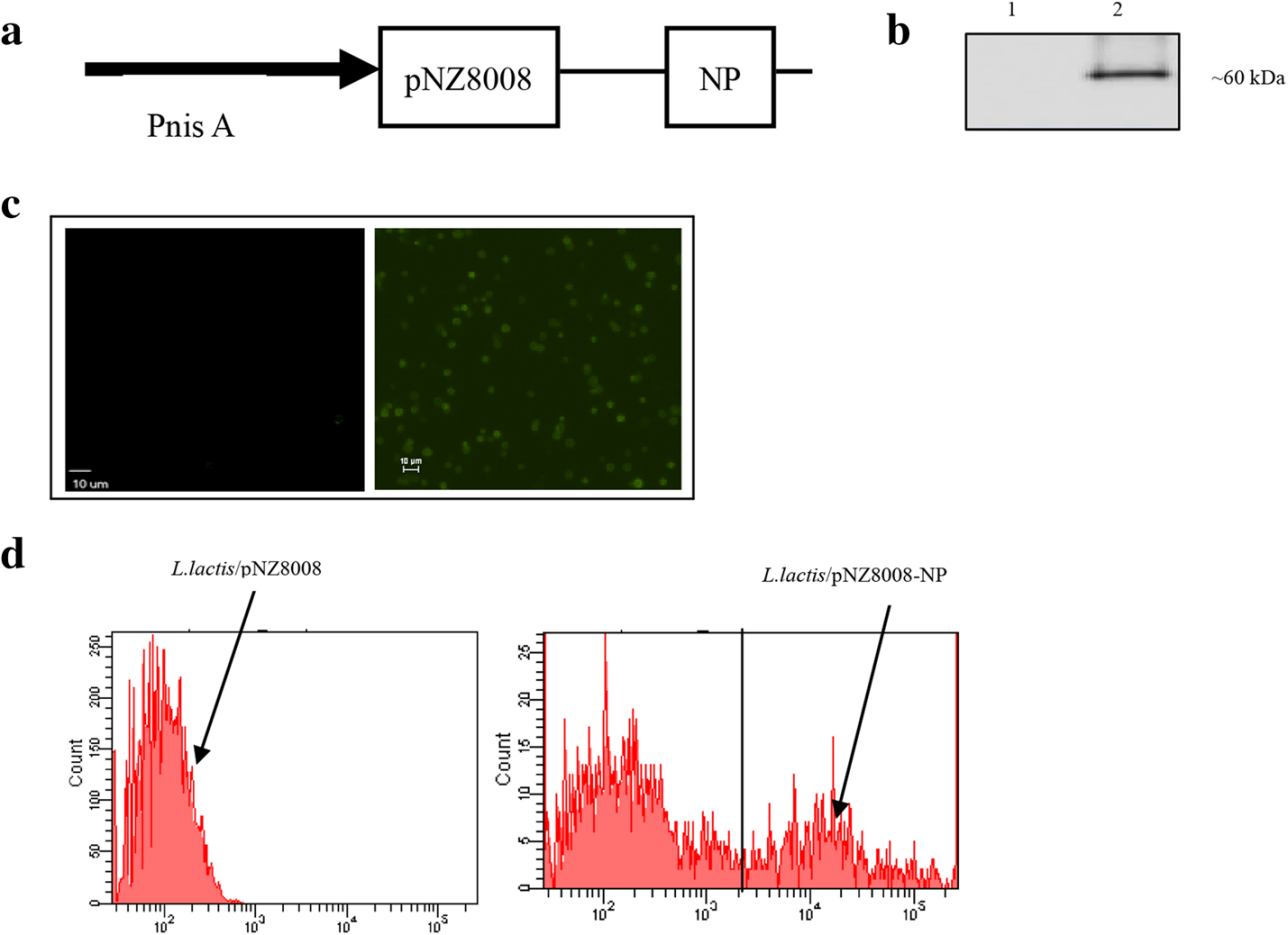
Expression of NP protein on *L. lactis*. **a** Schematic diagram of recombinant pNZ8008-NP. **b** Western blot analysis. *Lane 1 L. lactis*/pNZ8008; *Lane 2* *L. lactis*/pNZ8008-NP. **c** Immunofluorescence microscopy assay. *L. lactis*/pNZ8008 (*left*) and *L. lactis*/pNZ8008-NP (*right*) (magnification: ×1,000). **d** Flow cytometric analysis. 10,000 events were recorded.

Recombinant pNZ8008-NP plasmid was constructed (Fig. 1a). Expression of *L. lactis*/pNZ8008-NP was confirmed by Western blot analysis. A highly specific band (approximately 60 kDa) was observed in the *L. lactis*/pNZ8008-NP cells (Fig. 1b, Lane 2), whereas, there was no specific band shown in the *L. lactis*/pNZ8008 cells (Fig. 1b, Lane 1).

To test intracellular expression level of the NP protein, *L. lactis*/pNZ8008-NP cells were examined by immunofluorescence assay and flow cytometry analysis. As shown in Fig. 1c, d, specific cell staining was clearly evident when *L. lactis*/pNZ8008-NP cells were reacted with polyclonal mouse anti-NP antibody, confirming that NP protein was expressed on *L. lactis*. In contrast, there were no specific fluorescence signals detected in the *L. lactis*/pNZ8008 cells.

### Humoral and mucosal immune responses

**Fig. 2:**
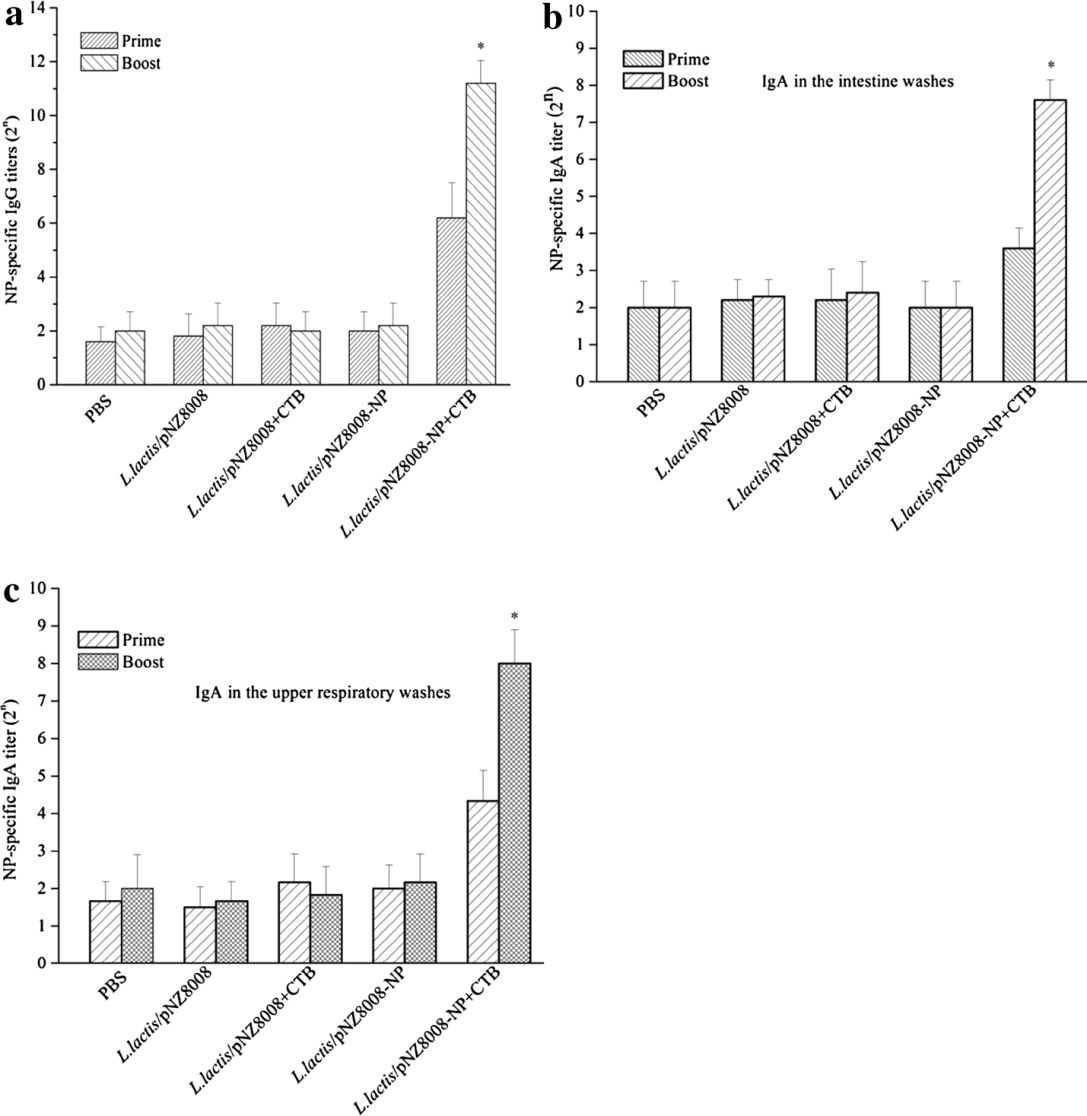
Humoral and mucosal immune responses elicited by *L. lactis*/pNZ8008-NP+CTB. ** a** NP-specific IgG antibodies in the sera (n = 19 mice/group); **b** NP-specific IgA antibodies in the intestine washes (n = 3 mice/group); **c** NP-specific IgA antibodies in the upper respiratory washes (n = 3 mice/group). Data are indicated as mean ± standard deviation (SD). *Asterisk* (*) shows statistical significance compared with PBS, *L. lactis*/pNZ8008, *L. lactis*/pNZ8008+CTB or *L. lactis*/pNZ8008-NP controls (*p* < 0.05).

NP—specific antibody responses were determined by ELISA. As shown in Fig. 2a, there were no significant sera IgG antibodies detected in all groups at day 16 after the initial immunization. However, only mice vaccinated orally with *L. lactis*/pNZ8008-NP+CTB could produce highly significant IgG titers at day 33 after the initial immunization. By contrast, there were still no significant changes in the PBS, *L. lactis*/pNZ8008, *L. lactis*/pNZ8008+CTB or *L. lactis*/pNZ8008-NP group.

Mucosal IgA antibodies were also measured in the intestine and upper respiratory washes (Fig. 2b, c), respectively. There were no significant IgA antibodies in all groups after the prime immunization (at day 16). Only *L. lactis*/pNZ8008-NP adjuvanted with CTB induced a higher level of IgA antibodies after the boost immunization (at day 33).

Collectively, these results demonstrate *L. lactis*/pNZ8008-NP alone is poorly immunogenic. However, the immunogenicity of *L. lactis*/pNZ8008-NP could be highly enhanced by the use of mucosal adjuvant CTB. Mice co-vaccinated orally *L. lactis*/pNZ8008-NP + CTB could produce significant NP-specific humoral immune responses, as well as mucosal immune responses.

### Cellular immune responses elicited by *L. lactis*/pNZ8008-NP adjuvanted with CTB

**Fig. 3:**
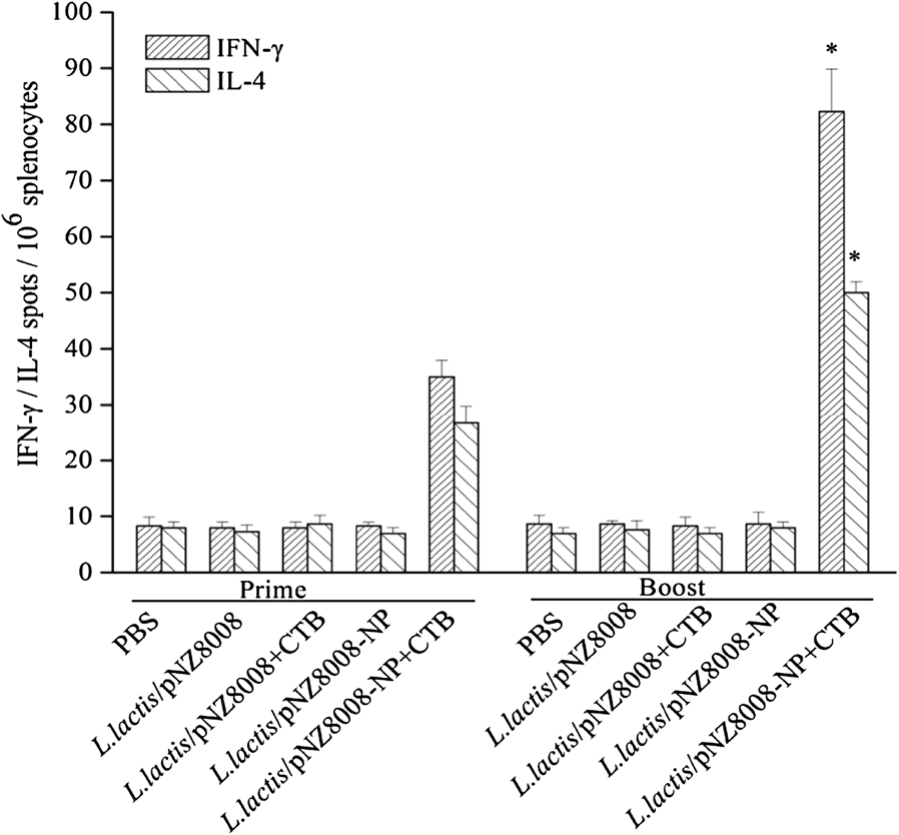
Cellular immune responses. IFN-γand IL-4 secreting spots were determined by ELISpot (n = 3 mice/group). Data are represented as mean ± SD. *Asterisk* indicates significant difference compared with PBS, *L. lactis*/pNZ8008, *L. lactis*/pNZ8008 + CTB or *L. lactis*/pNZ8008-NP controls (*p* < 0.05).

To test the cellular immune responses, INF-γ and IL-4 secreting cells were determined by ELISpot. As shown in Fig. 3, there were relatively low levels of INF-γ and IL-4 in all groups at day 16 after the initial immunization. However, at day 33, oral co-administration of *L. lactis*/pNZ8008-NP adjuvanted with CTB resulted in significantly higher levels of INF-γ and IL-4 producing cells. Overall, these results indicate that *L. lactis*/pNZ8008-NP adjuvanted with CTB can elicit significant cellular immune responses which include both Th1 and Th2 type immune responses with preferences of the Th1 immune responses.

### Cross protection against virus challenge

**Fig. 4:**
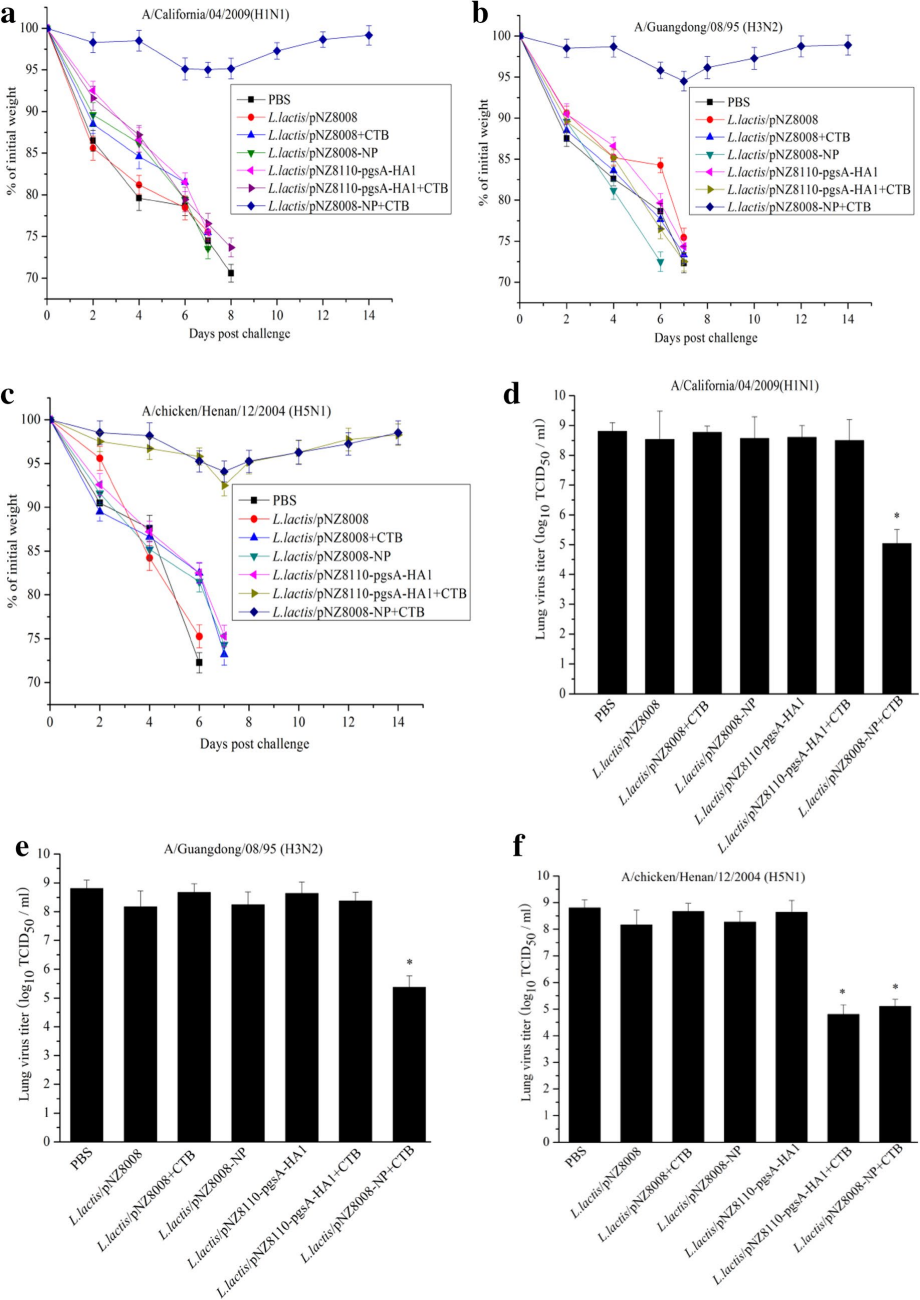
Cross-protection against divergent influenza A viruses. The results are expressed in terms of percent body weight (**a**–**c**) and lung viral titers (**d**–**f**). For a parallel experiment, *L. lactis*/pNZ8110-pgsA-HA1 alone or adjuvanted with CTB was used as a control for virus challenge. Data for lung viral titers (n = 3 mice/group) are represented as mean ± SD. *Asterisk* indicates significant difference compared with PBS, *L. lactis*/pNZ8008, *L. lactis*/pNZ8008+CTB, *L. lactis*/pNZ8008-NP or *L. lactis*/pNZ8110-pgsA-HA1 controls (*p* <  0.05).

**Fig. 5:**
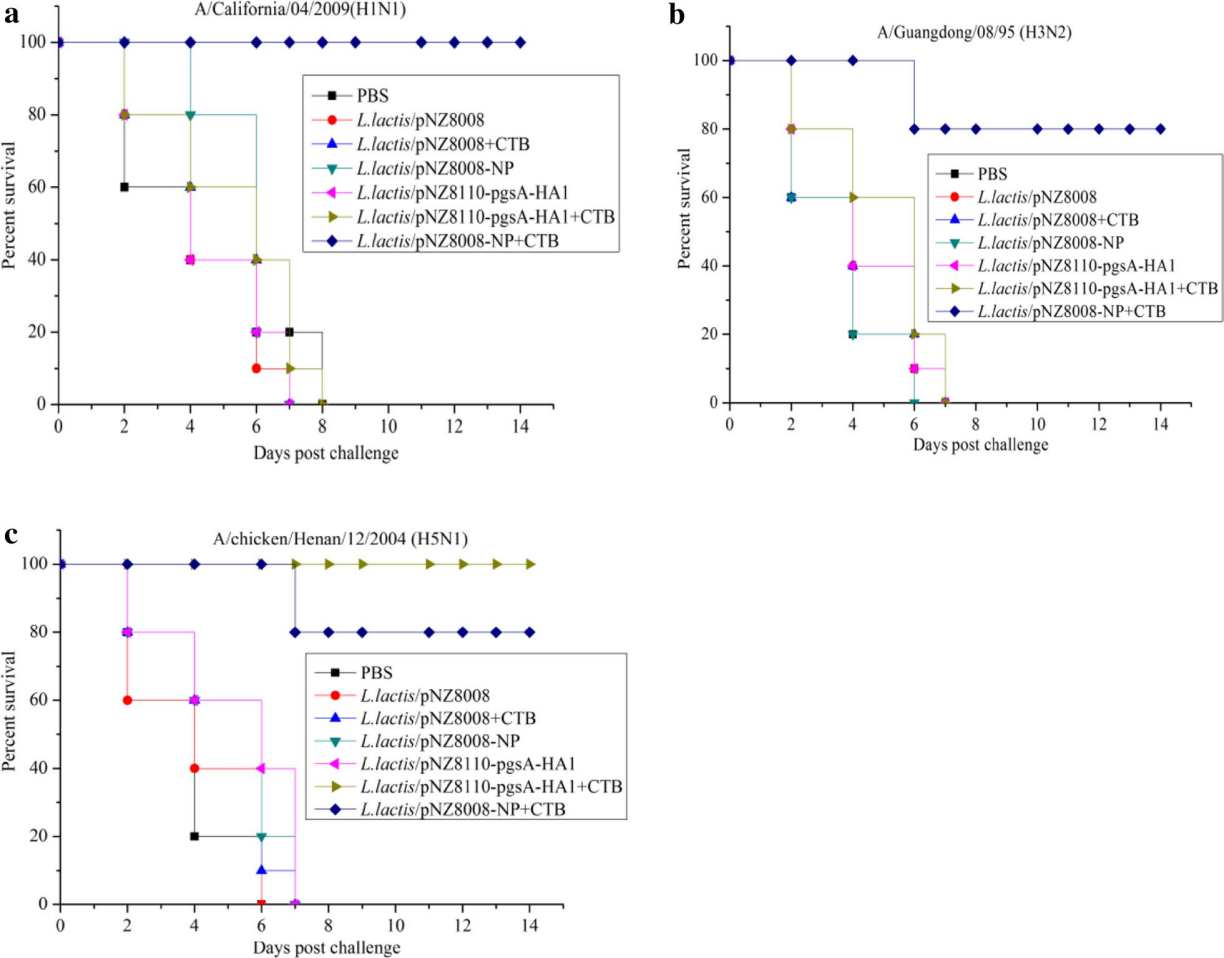
Percent survival. Two weeks after the last immunization, mice were intranasally infected with 20 µL of 10^4^ EID_50_ of lethal dose of A/California*/*04*/*2009(H1N1) (**a**), A/Guangdong/08/95 (H3N2) (**b**) or A/chicken/Henan/12/2004 (H5N1) (**c**). For a parallel experiment, *L. lactis*/pNZ8110-pgsA-HA1 alone or adjuvanted with CTB was used as a control for virus challenge. 10 mice/group were used to record survival rate.

To assess the cross protective immunity of *L. lactis*/pNZ8008-NP adjuvanted with CTB, we used *L. lactis*/pNZ8110-pgsA-HA1 adjuvanted with or without CTB as a control, all vaccinated mice were challenged with A/California*/*04*/*2009*(*H1N1), A/Guangdong/08/95 (H3N2) or A/chicken/Henan/12/2004 (H5N1), and monitored for 14 days. Interestingly, mice that received *L. lactis*/pNZ8110-pgsA-HA1+CTB showed a lower lung viral titer and slight weight loss against homologous H5N1 virus. Whereas, higher viral titers and significant weight loss against heterologous H1N1 or H3N2 were observed in the *L. lactis*/pNZ8110-pgsA-HA1+CTB group. Furthermore, *L. lactis*/pNZ8110-pgsA-HA1+CTB could provide 100% protection against homologous H5N1 virus infection, and show no protection against heterologous H1N1 or H3N2 virus challenge (Figs. 4, 5). The control groups that received PBS, *L. lactis*/pNZ8008 alone, *L. lactis*/pNZ8008+CTB, *L. lactis*/pNZ8008-NP alone or *L. lactis*/pNZ8110-pgsA-HA1 alone started to develop significant body weight loss, a higher lung virus titer and died at days 6–8 post challenge (Figs. 4, 5). In contrast, mice vaccinated orally with *L. lactis*/pNZ8008-NP + CTB were completely protected from homologous H1N1 virus challenge, and 80% protection against H3N2 or H5N1 virus challenge. All survived mice in the *L. lactis*/pNZ8008-NP+CTB group showed only mild weight loss, a lower lung virus titer and recovered by 14 days. Taken together, these results support that the immune responses induced by oral co-administration with *L. lactis*/pNZ8008-NP+CTB can confer cross-protection against divergent influenza A viruses in the mouse model.

## Discussion

Existing influenza vaccines provide effective protection against virus infection, but they provide strain-specific protection and need to be updated annually [4].Thus, there is a clear need for developing a universal influenza vaccine that would broadly protect against several strains [9]. In this regard, the highly conserved NP of the influenza A virus is an attractive candidate antigen for such a goal. Various systems have been applied to express NP protein [21–23, 29, 30], unfortunately, they show a limited cross-protection efficacy by intramuscular injection route. Mucosal administration represents an alternative approach to promote immune responses at the pathogen portal entry. The combination of a conserved influenza antigen with an efficient mucosal adjuvant could be a good approach to develop a universal influenza vaccine [21, 31, 32]. In this study, we chose *L. lactis* as a vaccine delivery vehicle, since it had a well-established safety profile. An exclusive plasmid of *L. lactis*, pNZ8008, could stably express viral antigen on *L. lactis*. NP expressed on *L. lactis* has shown a wealth of information regarding its characterization that can be reacted with monoclonal anti-NP antibody showing positive signals for Western blotting, immunofluorescence assay and flow cytometric analysis (Fig. 1). These data provide a possibility that the immunogenicity of NP based on *L. lactis* expression system can be further investigated via oral co-administration in the mouse model.

Increased serum levels of humoral immune responses were detected in mice vaccinated orally with *L. lactis*/pNZ8008-NP and CTB was used as a mucosal adjuvant (Fig. 2a). Although NP-specific IgG antibodies have no neutralizing activity, their importance should be highlighted. Several studies have shown that NP-containing immune-complexes released from infected cells could bind to Fc receptors on dendritic cells, thereby enhancing antigen presentation and subsequent viral clearance [33, 34]. Based on these findings, it was suggested that the capacity to stimulate anti-NP IgG may be a critical feature of a universal influenza vaccine [33]. Our study further testified that NP-specific antibodies could significantly contribute to protection against influenza virus infection.

Another prominent feature of *L. lactis*/pNZ8008-NP adjuvanted with CTB vaccine was the induction of significant NP-specific IgA responses in upper respiratory and intestine washes. This is particularly important since the influenza virus is a respiratory pathogen, colonizing trachea, bronchi and pulmonary alveoli as sites of viral replication [25]. It is tempting to speculate that this IgA might also contribute to cross-protection by a process known as intracellular neutralization which may play an important role in heterosubtypic immunity. Secretory IgA can be internalized within epithelial cells by the polymeric immunoglobulin receptor which would prevent viral assembly and neutralize viral infection [35]. Furthermore, the strategy for NP as an antigen is based on the elicitation of strong cell-mediated immunity rather than the induction of neutralizing antibodies [23]. In this study, an attractive feature of *L. lactis*/pNZ8008-NP adjuvanted with CTB is its extraordinary ability to induce a significant cellular response. Cytokine profiles of splenocytes from mice orally co-immunized *L. lactis*/pNZ8008-NP adjuvanted with CTB showed a secretion of INF-γ than IL-4 (Fig. 3), thereby suggesting a Th1 dominating immune response which may contribute to cross-protection.

It is well recognized that intranasal or oral delivery of antigen alone may not induce sufficient antibody response [25, 26]. In most of cases, the use of adjuvant will significantly increase the immunogenicity of antigen via mucosal administration [26]. Up to now, LTB and CTB are two strongest mucosal adjuvants, and have been verified by several studies [19, 21, 28]. Our previous studies have shown that oral administration of *L. lactis*/pNZ8110-pgsA-HA1 combined with 1 milligram of CTB provided protection against homologous H5N1 virus infection [19]. Although *L. lactis*/pNZ8110-pgsA-HA1 adjuvanted with CTB could provide protection against homologous H5N1 virus infection, it did not generate cross-protective immunity against heterologous H1N1 or H3N2 virus (Figs. 4, 5). Our current studies suggested that 5 × 10^11^ CFU of *L. lactis*/pNZ8008-NP adjuvanted with 1 µg of CTB, an optimal dosage for oral administration, could protect 100% mice against homologous challenge with H1N1 and 80% of mice against heterologous challenge with H5N1 or H3N2 (Figs. 4, 5). This means that the combination of *L. lactis*/pNZ8008-NP adjuvanted with CTB could provide the potential for the development of a novel universal influenza vaccine via oral co-administration route.

Taken together, the quality of the immune responses stimulated by oral co-administration with 5 × 10^11^ CFU of *L. lactis*/pNZ8008-NP adjuvanted with 1 µg of CTB vaccine was clearly demonstrated by the cross-protection against homologous and heterologous influenza virus challenge. This study clearly indicates that the immunogenicity of *L. lactis*/pNZ8008-NP would be greatly enhanced by CTB, and *L. lactis*/pNZ8008-NP adjuvanted with CTB is a promising universal influenza vaccine, which should be exploited to develop innovative vaccines against seasonal and pandemic influenza.

## Methods

### Construction of recombinant *L. lactis*/pNZ8008-NP

The NP gene (1,515 bp, GenBank: CY121683.1) of A/California/07/2009(H1N1) was PCR-amplified using pcDNA3.1- NP (kindly provided by St. Jude Children’s Hospital, Memphis, TN, USA) as a template and using the following primers with *Spe* I or *Hind* III site underlined (forward primer: 5′ CGCACTAGTATGAGTGACATCGAAGCCATGC 3′, reverse primer: 5′ CCGAAGCTTTTAACTGTACTCCTCTGCATTGTC 3′). The resulting *Spe* I/*Hind* III fragment was sub-cloned into pNZ8008 which was purchased from NIZO food research (Netherlands), and then electroporated into competent *L. lactis* NZ9000 which was a genetically modified host. The positive clone of *L. lactis*/pNZ8008-NP was selected as described previously [36]. *L. lactis* containing pNZ8008 without encoding NP gene (*L. lactis*/pNZ8008) was used as a negative control for the following tests.

### Western blot analysis

The expression of recombinant *L. lactis*/pNZ8008-NP was detected by Western blot analysis as described previously [36]. In a brief, 5 × 10^5^ cells of *L. lactis*/pNZ8008-NP pellets were mixed with 60 µl of 6 × loading buffer and boiled for 10 min, then run on SDS–polyacrylamide gel electrophoresis (SDS-PAGE) and transferred to nitrocellulose membrane (Bio-Rad, Hercules, California, USA). The membrane was blocked with 5% non-fat milk, and then incubated with a 1:500 polyclonal mouse anti-NP antibody (kindly provided by NIH Biodefense and Emerging Infections Research Resources Repository, Manassas, VA, USA), overnight at 4°C. Affinity-purified horseradish peroxidase (HRP)-conjugated anti-mouse IgG (Sigma-Aldrich Corporation, St. Louis, MO, USA) was used as second antibody. Finally, the proteins were visualized using enhanced chemiluminescence (ECL) reagents (GE Healthcare) according to the manufacturer’s instructions.

### Immunofluorescence assay

5 × 10^5^ cells of *L. lactis*/pNZ8008-NP were fixed with 4% paraformaldehyde for 10 min at room temperature (RT) and permeabilized with 0.2% Triton X-100 in PBS at RT for 10 min. The cells were subsequently blocked with 1% bovine serum albumin (BSA) in PBS for 30 min at RT, and then incubated with polyclonal mouse anti-NP antibody and followed by goat anti-mouse IgG antibody conjugated with fluorescein isothiocyanate (FITC) (R&D Systems, USA). Finally, the cells were visualized using a fluorescent Leica DM IL LED microscope (Leica, Wetzlar, Germany). *L. lactis*/pNZ8008 cells were used as negative controls.

### Flow cytometry analysis

5 × 10^5^ cells of *L. lactis*/pNZ8008-NP pellets were washed with cold washing buffer (1% BSA and 0.1% sodium azide in PBS), and then resuspended with 1% formaldehyde solution in cold wash buffer and fixed at 4°C in the darkness for 30 min, followed by incubation in 0.2% Triton X-100 in PBS for permeabilization at 37°C for 15 min. Following centrifugation, the cell pellet was resuspended in polyclonal mouse anti-NP antibody and incubated at 4°C for 30 min. The cells were washed and reacted with FITC-conjugated anti-mouse IgG secondary antibody at 4°C for 30 min in the dark. The stained cells were washed again and analyzed by flow cytometry analysis (BD FacsCalibur, San Jose, CA, USA).

### Vaccine, animals, immunization and sample collection

Recombinant *L. lactis*/pNZ8008 and *L. lactis*/pNZ8008-NP cells were adjusted to 10^12^ colony forming unit (CFU)/ml with sterile PBS, respectively. Specific pathogenic free (SPF) female BALB/c mice of 6 to 8 weeks of age were purchased from SLC Company (Shanghai, China). CTB was purchased from Sigma Chemical Company (St. Louis, MO, USA).

The mice (n = 25 per group) were vaccinated orally by a dosing needle with 500 µL of 10^12^ CFU/mL of *L. lactis*/pNZ8008 alone, *L. lactis*/pNZ8008-NP alone or adjuvanted with 1 µg of CTB on days 0, 1, 2 for prime immunization and days 17, 18, 19 for boost immunization. The oral dosage is equal to 5 × 10^11^ CFU of *L. lactis* vectored vaccine with or without 1 µg of CTB for each immunization. PBS was used as a negative control.

At day 16 and day 33 after the first immunization, blood samples were collected from the retro-orbital plexus. Sera were separated by centrifugation of blood at 2,000×*g* for 10 min and stored at −20°C until use. Intestines and upper respiratory (n = 3/group) were isolated from the vaccinated mice and washed with 500 µL sterile PBS, respectively, and stored at −20°C until use.

All animal immunizations were performed at biosafety level 2 (BLS-2) containment facilities complying with the Guidelines for Use and Care of Experimental Animals and were approved by the Animal Committee of the Institute of Nanchang University.

### Enzyme–linked immunosorbent assay (ELISA)

The NP-specific antibodies regarding IgG titers in the sera and IgA titers in the intestine and upper respiratory washes were determined by enzyme-linked immunosorbent assay (ELISA) using recombinant 2 µg/ml NP protein (kindly provided by NIH Biodefense and Emerging Infections Research Resources Repository, Manassas, VA, USA) as a coating antigen, as described previously [36]. End-point ELISA titers were expressed as the reciprocal of the highest sample dilution that yielded an OD ≥2 times above the mean value of the blank.

### ELIspot assay

To determine the levels of cellular immune responses, murine IFN-γ and IL-4 enzyme-linked immunospot (ELISPOT) kits (R&D Systems, USA) were used in this study, according to the manufacturer’s instructions. Splenocytes were isolated from the vaccinated mice at day 16 and day 33 after the initial immunization. Cells (5 × 10^5^ cells/well) were stimulated with 2 µg/ml of recombinant NP protein, and then incubated for 36 h at 37°C with 5% CO2. Cells were removed and the plates were processed according to the manufacturer’s instructions. Colored spots were counted with an ELISPOT reader (CTL S5 Micro Analyzer) and analyzed using ImmunoSpot image analyzer software v3.2 (CTL ImmunoSpot analyzer, OH, USA).

### Virus challenge

At day 35 after the initial immunization, all the vaccinated mice were challenged intranasally with 20 µL of 10^4^ EID_50_ of lethal dose of A/California*/*04*/*2009*(*H1N1), A/Guangdong/08/95 (H3N2) or A/chicken/Henan/12/2004 (H5N1) virus. The mice were monitored for 14 days and calculated with body weight loss and survival rate after post challenge. Lungs were isolated at day 5 post challenge. Virus challenge experiments must be strictly performed under the enhanced bio-safety level-3 laboratory (BSL-3). For a parallel experiment, *L. lactis*/pNZ8110-pgsA-HA1 alone or adjuvanted with CTB was used as a control for virus challenge [19]. Survival rate is less than 60% that was considered to be no significance.

### Lung viral titers

Lungs (n = 3 mice/group) were isolated at day 5 post challenge, as described previously [37]. In brief, 10-fold dilutions of lung homogenate supernatants were mixed with MEM including trypsin in 100 μL. Dilutions were added to 96-well U-bottom plates with 100 μL of Madin-Darby Canine Kidney (MDCK) cells at 2.5 × 10^6^cells/mL. The plate was incubated overnight at 37°C, and the medium was replaced with fresh MEM without trypsin. After 72 h incubation, 50 μL of 0.5% chicken red blood cells (CRBCs) was added to each well. The plate was incubated for 1 h at room temperature and recorded hemagglutination afterwards to determine 50% tissue culture infective dose (TCID50). TCID50 was then calculated by the Reed-Muench formula.

### Statistical analysis

The Student’s t test and one-way ANOVA were used for all statistical analyses. A *p* value less than 0.05 was considered to be significant.
